# Executive Function Level in Cadets’ Shooting Performance

**DOI:** 10.3390/ijerph19106007

**Published:** 2022-05-15

**Authors:** Dariusz Jamro, Grzegorz Zurek, Malgorzata Dulnik, Maciej Lachowicz, Dariusz Lenart

**Affiliations:** 1 Department of Physical Education and Sport, General Tadeusz Kosciuszko Military University of Land Forces, 51-147 Wroclaw, Poland; dariusz.jamro@awl.edu.pl (D.J.); dariusz.lenart@awl.edu.pl (D.L.); 2 Department of Biostructure, Wroclaw University of Health and Sport Sciences, 51-612 Wroclaw, Poland; mal.gosia009@gmail.com (M.D.); maciej.lachowicz@ideis.pl (M.L.)

**Keywords:** executive functions, shooting performance, gender differences, cadets

## Abstract

Executive functions (EF) are crucial to a person’s unique abilities, enabling one to achieve goals, adapt to new situations and manage social interactions. EF are also very important for the effective performance of military tasks including the shooting performance (SP) of soldiers. The aim of this study was to investigate the association of EF with SP and gender differences in the level of these traits among cadets of the General Tadeusz Kosciuszko Military University of Land Forces in Wroclaw i.e., 156 persons (19 females and 137 males). The level of EF and processes related to attention was measured with usage of the Color Trails Test (CTT-1 and CTT-2). SP was assessed on the basis of scores from four different small arms and rifle shootings at a fixed target and at emerging targets. The relations between explained and explanatory variables were assessed using Spearman correlation. The variation in the mean values of CTT scores and SP of men and women was compared using the Mann–Whitney U test for independent samples. The results of the present study did not reveal any significant differences between women and men in the level of EF and SP. The key finding of the present study is that the higher SP of males in all shooting events of the study and of females in pistol shooting were significantly correlated with higher executive functions.

## 1. Introduction

Executive functions (EF) are processes that allow the control of complex, conscious as well as intentional tasks. Thanks to them, humans are able to monitor and regulate their behaviour and perform so-called intentional activities [1]. These activities are particularly identified with the frontal lobe of the cortex and the cortical–subcortical neuronal network, but other areas are also involved in their regulation, i.e., the dorsolateral prefrontal cortex, anterior cingulate cortex or insula [2,3]. EF include cognitive abilities that enable a person to store information in working memory, inhibit automatic responses to stimuli (inhibitory control) and shift attention between related (attentional shift flexibility) but distinct aspects of a task or problem. Executive abilities, or cognitive control abilities, allow individuals to inhibit behaviors, focus attention and organize thoughts in the face of distraction, task complexity and stress [4].

The level of EF is positively influenced by sports participation. Studies of athletes’ brains show that enhanced neuronal networks and plastic changes are induced by the acquisition and execution of complex motor skills during daily intensive physical training. This training often requires rapid stimulus discrimination, specific attention and decision making. It is likely that the mode of neural modulation varies depending on the sport practised. Studies also confirm that open sports (e.g., basketball) can partially compensate for impaired executive control in people with limb impairments by supporting stability of motor responses and fostering flexibility of responses [5,6].

EF are also very important for the effective performance in military tasks performed by soldiers, including shooting abilities [7]. Military personnel are often required to engage in complex higher order cognitive tasks (working memory, inhibitory control, cognitive flexibility, planning, reasoning and problem solving) [8]. This occurs during physically demanding and stressful military exercises and especially in combat tasks under time pressure and threats to life and health. These tasks may include, but are not limited to, complex topographical orientation, decision making, memorising operational layouts and procedures or performing effective shooting tasks. The ability to maintain a high level of EF is certainly an important element of success among soldiers [9,10].

However, it is still unclear how and whether there are gender differences in the level of EF. Due to methodological variability and the involvement of multiple neuronal networks, it is not possible to make a simple, clear statement regarding differences between men and women in this area [11]. The literature provides divergent information regarding gender differences in CTT performance. Results from a study using the above instrument among 163 healthy participants aged 19–75 years showed a significant influence of age and education level on time to complete both parts of the CTT (higher age and lower education level contributed to slower time to complete both parts), while gender had no effect on time to complete Part B [12]. Data obtained from a U.S. standardized sample (1528 subjects, including 182 African-Americans and 292 Hispanics, ranging in age from 18 to 89 years) also confirm that the influence of gender on CTT scores is not significant; however, increasing age and lower education have been shown to adversely affect performance on this neuropsychological test [13]. Statistically significant differences between men and women were also not found by Konstantopoulos et al. and Hsieh and Tori [14,15]. Contrastingly, in other studies normalizing the Brazilian population, the data on CTT-1 and CTT-2 performance time by men and women were significantly different. Women presented higher mean scores (completion time), which corresponded to poorer performance [16]. Overall, gender does not seem to have a significant effect on CTT results.

Shooting efficiency, together with psychophysical efficiency, makes up the overall combat training of soldiers [17]. It involves accurate, i.e., effective shooting from individual weapons, and is a highly important skill, necessary to be mastered by every soldier. This activity, in connection with weapon handling, requires well-developed small motor skills [18,19]. Shooting is also a closed motor skill [20,21], which requires from a shooter highly developed anti-interference abilities, i.e., focus of attention and high mental intensity. Correct shooting actions (mainly pulling down the trigger tongue and maintaining a stable stance) are particularly associated with high demands on executive and inhibitory functions. In addition, the shooter updates his current behaviour with previous experiences in order to achieve a high score in the shooting task [22].

Often when shooting, shooters are exposed to stressful situations, e.g., during competitions, military exercises, difficult weather conditions or, finally, in warfare. In such situations, executive processes must occur at the highest level; this is necessary for the effective performance of the task [7,23,24]. During the firing of a shot it is also important to focus on the target, with simultaneous control of the body posture and such a positioning of the fingers to keep control over the trigger of the pistol. For this reason, both cognitive abilities and vigilance as well as appropriate motor skills are necessary to make an accurate shot [25]. For this to happen, alternating attention, which is one of the components of attention as a cognitive function, is also of considerable importance. Thanks to it, the shooter can control and coordinate all the activities mentioned above [26].

Various determinants of SP are sought in research; one of them is gender. Reports from the literature on gender differences in SP are divergent on this issue [27,28]. In a study by Kemnitz et al., female and male soldiers did not differ in their SP, although the men in the study sample had significantly less body fat, slimmer arms and were generally physically stronger than the female participants [29]. In the authors’ subsequent study, no significant differences were again found between male and female soldiers in accuracy or shooting precision, although it was initially suspected that this difference might be due to the size of the weapon (carbine weight and barrel length), which might be worse for women with less arm strength. As it has been proved, the above parameters proved to be significant for SP, however, irrespective of the gender [27].

Research on gender differences in SP has also been conducted in groups of athletes, where a good opportunity is a championship competition such as the European Championships. Mon-López et al. similarly showed no significant differences in SP in both rifles and air pistols. The lack of significant differences, according to the researchers, confirms that physical strength is an insignificant factor influencing performance in sport shooting, so the determinants of SP should be sought in other factors, perhaps in the shooter’s cognitive performance zone [30]. SP was also analysed after intensive exercise and caffeine consumption among reservists, where again no gender differences were found [31].

Gender differences in SP are extremely important in the police community where effective skill in the use of a personal weapon can often be decisive for one’s own and others’ safety. In fact, police officers may find themselves in a situation of direct danger on any duty day. Contrasting with the above literature reports, the results of studies in these populations indicate that male police officers shoot more effectively with handguns compared to females. The main factor attributed to the above differences is grip strength, which is potentially important to pistol shooting accuracy [28,32].

In another study, Johnson and Merullo showed that while men maintained the same accuracy during 3-h sentry sessions, accuracy in women deteriorated after 1.5 h. The shooting sessions consisted of detecting and shooting at targets that appeared infrequently [33]. In a recent study by Mon-López et al. comparing the performance of 704 shooters who participated in the recent World Shooting Championships, it was shown that men’s performance in pistol shooting was better than the performance of women. However, men and women performed the same in the overall analysis, while their performance differed by category and competition [34]. Researchers have also mainly focused their attention on rather obvious differences in physical factors such as upper body strength, grip strength, balance and coordination, important for high SP, neglecting the shooter’s cognitive abilities, which are potentially important [32].

To our current knowledge, the available literature does not provide sufficient information on the link between EF and SP. The issue of gender differences in the level of EF as well as differences in soldiers’ SP also requires supplementation and continuous analysis. The present study may therefore complement the literature with research in military populations. At the same time, the importance of conducting scientific research in the group of soldiers is emphasized, as the results may be particularly important from the point of view of benefits for national defence. The aim of this study was to investigate the association of EF with SP and gender differences in the level of these traits among cadets of the General Tadeusz Kosciuszko Military University of Land Forces in Wroclaw (MULF). We hypothesized that regardless of gender, cadets’ SP level would be related to their EF level.

## 2. Materials and Methods

The study group consisted of cadets—first-year students of the MULF. Cadets were in the course of their candidate military service and after 5 years of training they will start their professional military service. The main effect of education at MULF is the possession of appropriate knowledge and competences by graduates required to take up their first command positions in the officer corps.

**Figure 1:**
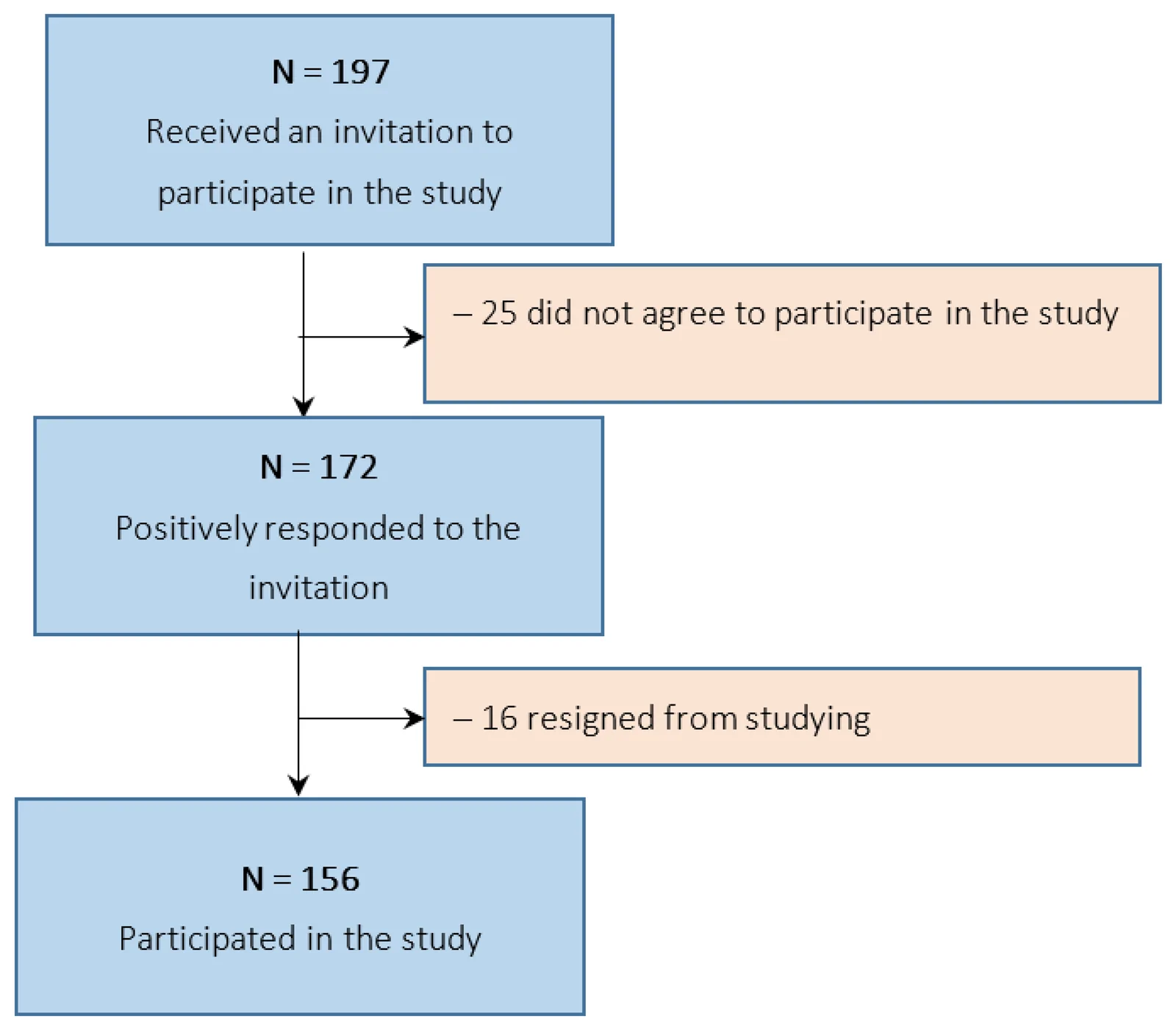
Flowchart of participant enrolment.

The criterium for inclusion in the study was obtaining promotion to the third semester of study. The respondents gave written, informed consent to participate in the study and were fully informed about the purpose of the study. Initially, 172 cadets fulfilling the study inclusion criteria were enrolled in the study. During the course of the study, 16 cadets dropped out of the study as they opted out of further military service. Ultimately, 156 individuals (19 females and 137 males), i.e., all cadets meeting the inclusion criteria, were included in the study. The study was conducted at the MULF in the period May–July 2021 (Figure 1).

## 3. Executive Functions

Measures of the level of EF and attention-related processes were the results of the Color Trails Test (CTT-1 and CTT-2). The Color Trails Test contains numbered coloured circles and language symbols with wide cross-cultural applicability (no language influence). The colours used in the CTT are universal. The visual stimuli in the CTT are circles with the numbers 1 to 25 written in the middle. Each circle is coloured yellow or pink. These colours are also seen by people with colour blindness. In the first part of the test (CTT-1), all odd numbers are in the pink circles and all even numbers are in the yellow circles. In the second part of the test (CTT-2), each number is printed twice, once in a yellow circle and once in a pink circle. The CTT sheets are printed on white paper measuring 21.59 × 27.94 cm. The universality of the CTT is due to the use of numbers and colours as symbols, with little involvement of speech and knowledge. The CTT is intended for adults (18+) and is designed to avoid making correct performance dependent on the knowledge of any alphabet and, to the greatest extent possible, on the influence of language [13,35].

The CTT-1 asks the participant to connect the circles in order from 1 to 25 with a rope as quickly as possible without taking the pencil off the paper. Before proceeding to the main task, the subject performs a trial task as fast as they can. The measure of the CTT performance is the time (in seconds) to correctly complete the task of connecting all circles from 1 to 25. The time is measured from the moment the test subject brings the pencil close to the first circle (starts the test). The stopwatch switches off as soon as the test subject touches the outer edge of the last circle with the pencil. In CTT-2, the tested person is asked to line up the numbered circles as fast as possible, taking into account the condition of colour alternation (pink circle 1, yellow circle 2, pink circle 3, etc.). As in CTT-1, a test task is performed before the main task.

The study was carried out among healthy individuals with no diagnosed clinical problems. Temporal indices of CTT performance are used to measure functions related to frontal lobe brain function. In interpreting the CTT results, a variety of processes related to attention and EF were examined, and, in particular, intentional search for material, sustained and metastable attention, sequential processing of information and monitoring of own behaviour were assessed [26].

The CTT was performed by a psychologist, took place in a lecture room, always under the same conditions (psychologist–subject) and at the same time of day. The time of task performance was measured with an accuracy of 1 s. The correctness and evaluation of the CTT performance was checked twice.

## 4. Shooting Performance

SP was assessed on the basis of the results of four different small arms and rifle shootings at fixed and emerging targets:

1. Rifle Shooting (RS)—consisted of shooting from a rifle in a lying position with the use of a stand on a stationary target 100 m away. The shooter had 5 cartridges and his task was to shoot with single fire. Accuracy and focus were important in this test. The score was determined by a number of points ranging from 0 to 50.
2. Shooting with a military pistol (PS)—this consisted of shooting from a military pistol in a standing stance at a stationary target 15 m away. The shooter had 5 cartridges; his/her task was to shoot with single fire; the shooting was performed with accuracy and focus. The score was determined by a number of points from 0 to 50.
3. Machine Pistol Shooting (MPS)—on the command “forward” the shooter marched or ran 10 m from the starting line to the firing line, assumed a shooting stance, prepared to fire and then began shooting. The first target was sighted 30 s after the command “forward” was given. The target appeared 5 times at a distance of 75 m. The time for each target to appear was 30 s. The interval between target appearances was 10 s. The score was determined by the number of hits on the target (score 5—4 hits, score 4—3 hits, score 3—2 hits).
4. Shooting from a rifle in a gas mask (RSG-M)—on the command “forward” the shooter marched or ran 10 m from the starting line to the firing line, assumed a shooting stance, prepared for shooting and then, after the first target appeared, started shooting. The first target appeared 30 s after the command “forward” and shooting was in short bursts while lying down with support. The target appeared 5 times at a distance of 100 m. The time taken for each target to appear was 30 s. Intervals between target appearances lasted 10 s. The shooter performed all actions in a gas mask. The score was determined by the number of hits on the target (score 5—4 hits, score 4—3 hits, score 3—2 hits).

All subjects had the same shooting experience resulting from the same military uniform training program. Every effort was made to ensure that shooting always took place in similar weather conditions and at the same time of day. The basis for conducting the tests was the consent of the Rector—Commandant of the university (No. 271 of 18 January 2021) and the consent of the Senate Committee on Research Ethics of the University of Health and Sport Sciences in Wroclaw (No. 2/2021 of 12 February 2021). All procedures performed in this study involving human participants were in accordance with the 1964 Helsinki declaration and its later amendments or comparable ethical standards. Written informed consent was obtained from all participants included.

## 5. Statistical Analysis

The collected results were subjected to statistical analysis. The normality of the distribution of individual variables was assessed using the Kolmogorov–Smirnov test. Of the variables analysed, the normal distribution was held by the shooting scores from all the shootings from the study in the women’s group and the scores from all the shootings and the time achieved in the CTT-1 in the men’s group. Non-normal distribution had CTT-1 and CTT-2 test scores in the female group and CTT-2 test scores in the male group.

The relationships between the explained and explanatory variables were evaluated using the Spearman correlation because not all variables analysed in the study had normal distributions. The correlation of each individual shooting score with CTT test scores was evaluated separately due to the different shooting conditions of the individual shooters and the different types of weapons. Different battlefield situations force a soldier to perform different shooting tasks, from shooting on full rest without additional stressors to shooting under conditions of very high physical effort, limited visibility or under time and danger regime. The different results of the correlation analysis are, in a way, a confirmation of the validity of this choice. This is because the authors wanted not only to see if there were relationships between EF and SP, but also to try to determine precisely whether, if such relationships existed, EF equally affected each shooting modality of the different weapons. The variation in mean values of CTT scores and SP of men and women was compared using the Mann–Whitney U test for independent samples.

Statistical significance was assumed at the level of *p* < 0.05 for all the applied tests. Calculations were performed using Statistica v. 13.1 software by StatSoft (Wroclaw, Poland)in the Biostructure Research Laboratory of the University of Health and Sport Sciences in Wroclaw, certified according to ISO 9001.

## 6. Results

The variation in the mean values of the tested variables by Mann–Whitney U test is presented in Table 1. As a result of the analysis, no significant differences in the mean CTT scores as well as in the SP of individual shooters were found between men and women. However, when comparing the mean scores of males and females, a lower arithmetic mean of the times achieved in CTT-1 and CTT-2 as well as higher SP in three out of four shooting events among females was observed. However, as mentioned above, these differences were statistically insignificant, so they can only be treated as a trend requiring possible further confirmation in subsequent studies.

The analysis of simple correlations between the level of EF and attention-related processes and the SP of male and female cadets is presented in Table 2. Of the four shooting events, only military PS was found to be statistically significantly negatively correlated with CTT-2 scores. Higher military PS scores were significantly associated with shorter CTT-2 performance in the female group. Such a high correlation coefficient (r = −0.76), indicates a very strong interdependence of the studied variables. The remaining correlations in the female group were statistically insignificant.

In the men’s group, higher SP in RS was significantly correlated with shorter CTT-1 time. This is evidenced by the negative correlation coefficient, but the strength of these correlations was weak (r = −0.23). The results from the other shootings in the male group were not significantly correlated with the time achieved in the CTT-1 attention test. The correlation results, however, revealed mutual correlations between all the shooting results from our study and the time achieved in CTT-2. SP appeared to be significantly negatively correlated with CTT-2 performance, so in the male group, higher SP was significantly correlated with shorter CTT-2 performance time. Strong correlations occurred between RS (r = −0.47) and PS (r = −0.51) and CTT-2 performance. The correlation between MPS performance and CTT-2 time was at the average level (r = −0.39). A weak but also significant correlation occurred between RSG-M scores and time in the CTT-2 (r = −0.18).

## 7. Discussion

EF help to resist strong internal tendencies and external stimuli, including controlling attention, behaviour, emotions and thinking, and focusing on ongoing action to make appropriate behavioural decisions [36]. The results of our study did not reveal significant differences between women and men in the level of EF. The literature provides divergent information regarding gender differences in CTT performance. Statistically significant differences were not found by Konstantopoulos et al. or Hsieh and Tori [14,15]. Different data were presented by Rabelo et al., who obtained a statistically significant difference in favour of men [16]. In another study, Gaillard et al. conducted a systematic review of the literature aimed at summarising the current evidence on sex differences in three domains of EF: performance monitoring, response inhibition and cognitive set-shifting using functional neuroimaging tools (fMRI, PET, EEG and NIRS). A meta-analysis of 21 studies, involving a total of 677 women and 686 men, indicated that due to methodological variability and the involvement of multiple neuronal networks, it was not possible to provide a simple, binding statement on the differences between men and women in levels of EF. However, there is now evidence of sex differences in the neural networks underlying all EF considered in this review, suggesting that men and women use different strategies depending on the demands of the task. However, functional neuroimaging, although a highly detailed and valuable study, may not be sufficient to identify sex differences in the level of EF, so work using neuropsychological tests such as the CTT may complement the above studies [11].

Comparative value with the results of our own research in the context of gender differences in the level of EF is provided particularly by data from standardisation trials for the CTT. The results of the American standardisation trial among 1531 individuals (male = 1345, female = 183) confirmed that, regardless of age group, no gender factor influence on CTT scores was found. It should be noted that women in this sample constituted 12% of the total study population, as in our study [13].

On the other hand, in other studies normalizing the Brazilian population, the data on CTT-1 and CTT-2 performance time by men and women were significantly different. Women presented higher mean scores (completion time), which corresponded to poorer performance [16]. A subsequent study among a Greek population of healthy subjects (men = 79, women = 84) showed little effect of gender on CTT-1 performance time (women performed relatively worse compared to men). This contradictory finding compared to the results of our own study may be attributed to the fact that women in the above studies had lower levels of education compared to men [14]. The literature confirms that education has a significant effect on the level of EF tested by the CTT test [16,37]. In our study, men and women represented the same level of education resulting from the same educational program. It should additionally be noted, as is usually the case with normative research in studies, the participants were from a wide age range, while in our study the study group was a first-year military community in the age range (20–26 years).

Studies in military settings confirm that men perform better in military tasks, especially those requiring prolonged use of strength and endurance [38,39]. However, it is still not entirely clear whether gender differences are equally obvious in such specific tasks as shooting. Shooting is one of the most important skills indicative of a soldier’s preparedness, which translates into the combat capabilities of a military unit. Since women began to join the ranks of armies in various countries, a discussion has begun about their role in combat operations. Researchers began to focus their attention mainly on rather obvious physiological differences, neglecting cognitive abilities and various elements of combat training including shooting efficiency. According to our current knowledge, the literature on the subject is quite poor in cross-gender comparative analysis of soldiers in terms of SP. In our study, no significant differences in shooting efficiency were observed between men and women. Despite the fact that in three out of four shootings women achieved slightly better results, these results were not statistically significant. Similar results were observed in their study by Kemnitz et al., who evaluated the effect of gender on shooting accuracy in a group of 15 male and 13 female soldiers. The Noptel simulator was used to assess accuracy (distance of shots from the centre of the target) and precision (distance of shots from each other regardless of distance from the centre of the target). As in our own study, no significant differences were found in any of the measures of SP according to gender. Although the above results confirm the reports from our own study, it should be noted that it was conducted under different shooting conditions. The main difference consisted in shooting from a simulator, whereas the shooting in our study took place on an open range; additionally, different weapons were used and different targets were shot at different distances. However, it can be concluded that women and men do not differ in SP regardless of the different shooting conditions [27].

In another study involving 292 shooters who competed in the 2016 and 2018 European Championships, men and women shot equally well with rifles, and although men’s average pistol scores were higher than women’s, the difference was not statistically significant. It was concluded that in sports where physical strength is a less important factor, as in the case of sport shooting, the rules should be revised for greater gender equality [30]. The above results are consistent with those of our own study, but the fundamentally different shooting conditions and the different type of weapons should be noted. In the study cited above, participants dressed in a special shooting suit shot with an air rifle, whereas in our study soldiers shot with firearms in tactical gear. In contrast, a study by Goldschmied et al. found no differences in the performance of men and women in shooting either an Olympic air rifle or a 22 caliber rifle in shooting competitions. The authors justify this on the grounds that “in shooting, the physical demands on athletes are relatively low”. The study by Goldschmied et al. corresponds with the results of our own study confirming the lack of significant differences in SP among both shooters with low shooting experience and at the highest competitive level [40].

Interesting results were presented by Vučković et al., who, in a group of male and female police officers, determined the effectiveness of a basic training program in the use of small arms. During the three stages of the study, i.e., at the beginning, in the middle and at the end of the shooting training, significant differences in SP between men and women emerged only at the beginning of the training. Moreover, the same shooting training program increased the final SP by 136.43% among women, while the increase was 45.69% among men. Thus, the above results confirm that women do not differ from men in SP in both long and short arms [41].

However, some studies report gender differences in small arms SP. Anderson et al. found that male police officers performed better with pistols than female police officers [32]. Similar results were obtained by Copay et al., who observed that males performed better than females in shooting 9 mm, 0.40 inch (.40 Smith & Wesson) and 0.45 inch (.45 Automatic Colt Pistol) pistols [28]. The different shooting effects are attributed to the difference in grip strength, although its effect on SP was small. The results of our own study also do not agree with other studies that found that men performed better than women in shooting with 22 caliber rifles at a distance of 50 metres [42] and in military conditions with rifles [33]. The military study, unlike our own work, was conducted on a Weaponer simulator. The results of this study showed that during the first 1.5 h of guard duty, women shot as accurately as men. It was only after 1.5 h that their rifle shooting accuracy deteriorated and they did less well with accurate shots on target, with no deterioration in reaction time in the form of detecting the target and firing the shot. According to the authors, this difference may be due to gender differences in hand stability or to possible weaker upper body strength (causing greater fatigue after a longer duration of the combat task). However, both hypotheses are speculative and require further research.

It seems crucial to note in our study that the higher shooting scores achieved by males in all shooting tasks of the study and by females in PS were strongly significantly associated with higher EF. The strongest significant correlations occurred in carbine and pistol shooting in the male group, which was probably caused by the shooting conditions consisting in firing at a fixed target with accuracy and focus without a time regime and on full rest. A weaker correlation occurred in the machine pistol shooting, in which there was another stress factor of moving quickly to the shooting position and the target appearing in a limited time, which was undoubtedly a big limiting factor for the shooter [7]. These factors revealed that in more dynamic shooting, further variables related to SP, such as physical fitness, are likely to emerge alongside EF.

The weakest significant correlations occurred in shooting after rapid movement to the shooting position, in shooting under the regime of target appearance time and probably due to the gas mask worn during all activities. The gas mask makes it very difficult to fire effectively; it sometimes fogs up during physical exertion and shooting, which impedes the visibility of the target and aiming devices, has a limited field of vision and significantly impedes the acquisition of a comfortable and appropriate shooting stance (head position in relation to the weapon) [43].

The strongest significant effect of EF on SP was revealed in military pistol shooting in both the male and female groups. This shooting was performed on accuracy and focus in a standing stance. It was by far the easiest or one of the easiest shootings of the test, as the distance to the target was only 15 m and the target at which the fire was conducted was the same as in the carbine shooting at a distance of 100 m. Of course, small arms were fired, but the very short distance to the target made it more “forgiving” of possible shooter errors [30]. The lack of significant correlation between CTT-1 performance time and SP, except for carbine shooting in the group of males where the strength of correlation was weak anyway (r = −0.23), clearly indicates that the second part of CTT, i.e., CTT-2, should be used to study complex performance functions with a potential relationship to the SP of soldiers [13].

Since males and females did not differ in their level of SP, and among males all shootings were positively associated with shorter CTT-2 performance, the lack of significant correlations in three out of four shootings with CTT-2 performance time in the female group can be explained by their small numbers. It can also be assumed that the remaining correlations would probably have been revealed if the number of women had been similar to the number of men. However, the study included all women who studied at MULF.

The literature recognizes that military performance depends on high levels of cognitive, EF, especially during heavy physical exercise [7]. Associations of some EF with SP among soldiers were found, among others, by Hillman et al. They examined EEG activity during the preparation period between executed and rejected shots to better understand the attentional processes associated with the pre-shooting state. As in our study, they recognised the large role of attention in the complex process of firing a shot. Additionally, it was found that the decision to reject a shot appears to be characterised by a misallocation of neural resources associated with task performance. The study, unlike our own research, was conducted on a group of skilled sharpshooters who performed shooting at much greater distances than cadets, so comparisons should be made with caution [25].

Additionally, the results of our own research confirm the recent study by Shao et al. in which it was reported that self-control (as one of the elements of EF) during the performance of closed motor tasks in the environment determines that shooters have a higher anti-interference ability. This ability, in turn, boils down to the deliberate and active selection of specific data from the environment in shooting, i.e., focusing attention only on selected important information while ignoring other distracting stimuli from the environment [22]. Furthermore, the work of Sattlecker et al. focused on athletes at World and European Cup level in biathlon showed that postural balance and rifle stability play a key role in this sport [44]. Studies on such a skilled shooting group prove that shooters must be strongly engaged with the target while aiming and working on the weapon (attentional control) and constantly monitoring their behaviour and controlling their emotions to minimise the risk of making a mistake. On the other hand, when a mistake is made, they should make sure it has as little impact on the result as possible. For example, if a mistake occurs in the form of a “missed shot”, i.e., a trigger pull that is too fast, the shooter should quickly and accurately identify the problem, which is related to the control of emotions, and then make the appropriate correction and prevent a similar mistake from being repeated in the future (behavioural control). Comparing the above study with the results of our own research, it should be noted that the shooting was performed indoors at a distance of 50 m with a specialised air rifle fitted individually to each athlete without any additional physical load. Military shooting, on the other hand, usually involves an additional mental and physical load, so the greater use of EF in the form of behavioural control, emotions, focus of attention and the ability to eliminate external stimuli may be key determinants in SP.

Our results also show that the strength of the correlation between SP and the level of EF decreased with the more difficult level of shooting (shooting after a run-in and in a gas mask). This was probably related to a decrease in the level of EF under the influence of a greater external stressor such as a short and quick change in shooting stance, a gas mask and targets appearing in a limited time. The above results correspond with those of previous studies on EF at specific exercise intensities. Labelle et al. observed a significant decrease in EF at both 60% and 80% of peak power output [45]. Furthermore, Lo Bue-Estesa et al. confirmed that EF decreased during post-exercise assessment [43]. Additionally, the results of our own study are consistent with the results of a study among Reserved Officer Training Corps (ROTC) cadets, in which it was proven that high-intensity exercise decreases EF [7].

The results of our study confirm the hypothesis that gender is not a significant factor that affects the level of EF and the SP of soldiers. A higher level of EF has a significant effect on higher SP of male soldiers in all types of shooting from the present study with both carbines and pistols. However, among females, higher EF has a significant effect on higher pistol SP.

Summarizing the results of our own research and from the literature reports on gender differences in SP, it can be noted that there are still many unexplained issues. Noticeable discrepancies may be a result of differences in the way SP is measured in individual studies, the degree of shooting difficulty and natural somatic and motor differences. Therefore, it seems reasonable to systematically examine the SP of soldiers in a way that most accurately reflects the requirements of the population of interest, in particular, the study of soldiers’ actions in real combat and training ground conditions. The level of shooting training of an individual soldier and his cognitive efficiency (especially of a commander–leader) is of great importance for the combat potential of the armed forces and should be a key aspiration of each military unit. The results of the conducted research as well as many other works confirm the necessity of placing great emphasis on the cognitive preparation of a modern soldier, in particular aimed at the development of EF skills. They have a significant impact on the level of a soldier’s shooting efficiency.

## 8. Limitations, Strengths and Future Research

The upper or lower values for some variables are due to hazard. This is the meaning of statistics when *p* < 0.05. Significant relationships and differences were shown on a sample with small numbers of women relative to men. We saw significant relationships between the variables studied, or lack thereof, but our evidence is weak because we showed them on a small number of subjects relative to men. Thus, we are not entirely sure that what we see is not a coincidence (which is always easier the smaller the sample), and that we will see a strong relationship again when we repeat this experiment; so more research is needed. In contrast, we saw strong evidence in statistically significant correlations in a large group of men.

Due to the simplicity of application in a military setting, the level of EF and processes related to attention were assessed using the Color Trails Test (CTT-1 and CTT-2). Despite the great popularity of neuropsychological tests for the assessment of EF in research (CTT, Wisconsin Card Sorting Test, Stroop Test) [46,47], it is now also possible to assess EF using neurophysiological methods such as visual or auditory evoked potentials and gamma oscillation. Increasingly, high-tech neuroimaging methods are also being used for neurocognitive measurements.

There is a lack of research in the literature on the relationship between EF and cadet SP, so this opens up useful new areas of research, particularly for researchers from the military community. Further research would be worthwhile to consider other factors influencing SP (e.g., type of shooting training, kinematic analysis of shooting stance and weapon stability, physical fitness) potentially important for the level of shooting training. Moreover, a lot of valuable information may be provided by the results of future research on dynamic SP under conditions of higher physical and mental fatigue in both male and female groups, as they will be more relevant to the realities of combat operations.

## 9. Conclusions

EF are crucial to unique human abilities including, as it turns out, soldiers’ SP. A more thorough analysis of the components of EF may help to develop targeted interventions to improve them. This knowledge should be of particular interest to researchers in the uniformed services community and those professional groups where weapons are the primary tool.

The results of our own research indicate that female cadets represent a similar level of EF as well as SP; therefore, the load and evaluation methods in training should not be differentiated by gender. However, the problem of gender differences in specific professions such as uniformed services remains open and requires further detailed analysis.

## Figures and Tables

**Table 1:** Variation in mean CTT scores and shooting performance by Mann–Whitney U test for independent samples between men and women. Variation coefficients in bold are significant with *p* < 0.05.

Variable	Men (*N* = 137)			Women (*N* = 19)			U Mann–Whitney Test
	$\bar{x}$	sd	v	$\bar{x}$	sd	v	*p*
Age (years)	21.03	1.31	6.22	21.15	1.08	5.11	0.3683
Body height (cm)	179.17	5.58	3.11	167.27	4.40	2.63	**0.0000**
Body mass (kg)	76.82	7.69	10.01	61.83	3.41	5.52	**0.0000**
CTT-1 (s)	31.12	7.99	25.66	28.58	10.68	37.37	0.2105
CTT-2 (s)	59.84	12.54	20.95	58.74	11.11	18.92	0.8550
RS (score)	36.64	5.90	16.10	36.16	5.74	37.37	0.4255
PS (score)	34.53	8.63	24.99	35.53	9.32	18.92	0.5576
MPS (grade)	4.46	0.84	18.84	4.89	1.13	15.87	0.0651
RSG-M (grade)	4.39	0.93	21.08	4.79	0.32	26.24	0.0745

RS—rifle shooting, PS—pistol shooting, MPS—machine pistol shooting, RSG-M—shooting from a rifle in a gas mask.

**Table 2:** Spearman correlation results between the study variables. Correlation coefficients in bold are significant with *p* < 0.05.

Variable	Men		Women	
	CTT-1	CTT-2	CTT-1	CTT-2
RS	**−0.23**	**−0.47**	−0.20	−0.35
PS	−0.10	**−0.51**	−0.21	**−0.76**
MPS	−0.13	**−0.39**	−0.16	0.28
RSG-M	−0.03	**−0.18**	−0.11	−0.28

RS—rifle shooting, PS—pistol shooting, MPS—machine pistol shooting, RSG-M—shooting from a rifle in a gas mask.

## Data Availability

The datasets used and/or analysed during this study are available from the corresponding author on reasonable request.
